# Review and Meta-Analyses of TAAR1 Expression in the Immune System and Cancers

**DOI:** 10.3389/fphar.2018.00683

**Published:** 2018-06-26

**Authors:** Lisa M. Fleischer, Rachana D. Somaiya, Gregory M. Miller

**Affiliations:** ^1^Department of Pharmaceutical Sciences, Northeastern University, Boston, MA, United States; ^2^Department of Chemical Engineering, Northeastern University, Boston, MA, United States; ^3^Center for Drug Discovery, Northeastern University, Boston, MA, United States

**Keywords:** PBMC, platelet, granulocyte, lymphocyte, B-cell, T-cell, astrocyte, microglia

## Abstract

Since its discovery in 2001, the major focus of TAAR1 research has been on its role in monoaminergic regulation, drug-induced reward and psychiatric conditions. More recently, TAAR1 expression and functionality in immune system regulation and immune cell activation has become a topic of emerging interest. Here, we review the immunologically-relevant TAAR1 literature and incorporate open-source expression and cancer survival data meta-analyses. We provide strong evidence for TAAR1 expression in the immune system and cancers revealed through NCBI GEO datamining and discuss its regulation in a spectrum of immune cell types as well as in numerous cancers. We discuss connections and logical directions for further study of TAAR1 in immunological function, and its potential role as a mediator or modulator of immune dysregulation, immunological effects of psychostimulant drugs of abuse, and cancer progression.

## Introduction

The Gs-linked G-protein coupled receptor Trace Amine Associated Receptor 1 (TAAR1) is a target for a wide variety of agonists including endogenous amines and amphetamine-like drugs of abuse. Endogenous agonists include common biogenic amines as well as trace amines (TAs) (Borowsky et al., 2001; Bunzow et al., 2001). TAs are present in the mammalian nervous system at levels much lower than those of common biogenic amines and include β-phenylethylamine (β-PEA), tyramine, octopamine, tryptamine, and thyronamine (Boulton, 1976; Juorio, 1982; Burchett and Hicks, 2006). TAs resemble common biogenic amines in terms of subcellular localization, chemical structure, and metabolism (Borowsky et al., 2001; Lindemann and Hoener, 2005). TAAR1 is also a target of drugs of abuse including methamphetamine, amphetamine, and 3,4-methylenedioxymethamphetamine (MDMA) (Bunzow et al., 2001). Unlike common biogenic amines, both TAs and amphetamine-like drugs show greater selectivity for TAAR1 relative to other aminergic receptors, which we have hypothesized to underlie both signaling regulation and dysregulation related to imbalances between these signaling molecules in psychiatric conditions and in addiction (Xie and Miller, 2008). Aminergic neurotransmitter imbalance is implicated in several neuropathological conditions and therefore the major focus of TAAR1 research has been its role in monoaminergic regulation, drug-induced reward, and psychiatric conditions (Miller, 2011). Less studied is TAAR1 expression and functionality in immune cells, which is the subject of this review.

The immune system is a complex collection of organs, tissues, and cells that serve to protect the host organism from pathogens. In its simplest form it can be subdivided into the innate and adaptive immune systems based on the temporal sequence of the immune response (Janeway, 2001). While immune cell interactions are extremely complex and the lines separating branches of immunity are ambiguous at best, for the purpose of this review we will follow this basic dichotomy. Here, we review the immunologically relevant TAAR1 literature and incorporate open-source expression and cancer survival data as a mode of observational insight into physiologically-relevant topics of interest not only to the TAAR1 research community, but also to other life science investigators. We mined the NCBI Gene Expression Omnibus (GEO) profiles database (Barrett et al., 2013) which contains gene expression profiles from curated GEO Datasets (GDS) to find evidence of TAAR1 expression across various immune cell types and cancers. We then make connections and suggest logical directions for further study of TAAR1 in immunological function. We also provide complete references containing queryable NCBI GEO Profile ID and GDS accession numbers.

## Methods

### Microarray datasets

Microarray datasets containing normalized counts corresponding to the TAAR1 gene transcript were obtained through BioGPS, RefDIC, and NCBI GEO. Normalized counts were log2 transformed and visualized in R.

#### BioGPS

Datasets for TAAR1 expression were extracted from http://biogps.org using the search function to query for “TAAR1.”

#### RefDIC

Transcriptomic profiling data of immunologically relevant cell types was obtained from the Reference Database of Immune Cells (RefDIC, Hijikata et al., 2007). Datasets were extracted using the “Expression profile” function (available at http://refdic.rcai.riken.jp/profile.cgi) utilizing the microarray profile type and using the official gene symbol “TAAR1.” The human and mouse TAAR1 gene was selected (ID 134864 and 111174, respectively). Queries for various immune cell types were conducted using a text search within the “Change Dataset” function.

#### NCBI GEO

The NCBI Gene Expression Omnibus (GEO) includes the GEO Profiles database (Barrett et al., 2013) which contains gene expression profiles from curated GEO Datasets (GDS) that are searchable by gene identifier and by keywords. The GEO Profiles database was probed by cell type using the advanced search builder function to build a query containing cell types of interest. Briefly, to extract all GEO Profiles containing data for TAAR1 RNA expression in astrocytes, for example, a search was conducted using the terms “TAAR1 (AND) ASTROCYTES.” Other immune cell types were similarly queried using this search method.

GDS accession numbers, literature references, and Profile ID numbers associated with their respective GEO Profiles are included in tables to allow for direct access to the expression data discussed. Individual GEO Profiles discussed in this review may be freely accessed at https://www.ncbi.nlm.nih.gov/geoprofiles/ by entering the GEO Profile ID provided in the text search box. Similarly, entire GEO Datasets may be accessed in the same manner at https://www.ncbi.nlm.nih.gov/gds. As the microarray technology used to produce GEO Profiles commonly utilizes an algorithmic detection call to determine cutoffs for positive signals, some GEO Profiles contained samples in which the TAAR1 expression signal was below the cutoff for expression, and those samples are denoted as “Below cutoff” in tables. GEO Profiles where not all but at least one sample was positive for TAAR1 expression are denoted as “Partial” in tables, and GEO Profiles where all samples were negative for TAAR1 expression signal are denoted as “Negative.”

When available, statistical analysis within a dataset was carried out using the Analyze Dataset tool located at the bottom of the dataset of interest's page.

### Tissue protein expression datasets

#### Human protein atlas

Antibody-based protein expression data is freely available online from the Human Protein Atlas (HPA) at www.proteinatlas.org (Uhlén et al., 2015; Thul and Lindskog, 2017). This database was generated by probing various human tissues for all protein-coding genes and is cataloged in a searchable module, allowing for tissue-specific exploration of expression patterns. For antibody-based data, the gene of interest is assigned an expression level based on staining intensity and fractional quantity of stained cells in a sample. TAAR1-specific results can be viewed directly at https://www.proteinatlas.org/ENSG00000146399-TAAR1/tissue.

### RNAseq datasets

#### BioXpress

A text query for the HGNC gene symbol “TAAR1” was made using the mRNA transcript search tool available from https://hive.biochemistry.gwu.edu.

#### cBioPortal

Processed RNAseq data representing 41,907 RNAseq samples across 171 cancer studies was obtained from cbioportal.org using a text search for “TAAR1” in the gene query tool.

#### Catalog of somatic mutations in cancer (COSMIC)

A gene query for “TAAR1” was carried out using the Genome Browser tool available at https://cancer.sanger.ac.uk/cosmic/browse/genome.

#### Cancer RNAseq nexus (CRN)

Datasets were obtained through a text search for “TAAR1” at http://syslab4.nchu.edu.tw/CRN/.

#### The cancer genome atlas (TCGA) transcriptomic profiling

RNA-seq expression profiles for TAAR1 were obtained from the NIH National Cancer Institute Genomic Data Commons (GDC) Data Portal at https://portal.gdc.cancer.gov/. RNA-seq datasets for the TCGA cancer studies discussed can be accessed from the link above by navigating to the “Exploration” tool, then selecting “Genes,” entering “TAAR1” in the search bar, then navigating to “View Files in Repository” and finally selecting “Transcriptome Profiling.” Cancer RNA-seq expression dataset names begin with the prefix “TCGA-” followed by the abbreviation for the appropriate cancer type. Data can be downloaded as.TXT files and are searchable by text to identify data specific to TAAR1.

#### Arrayexpress

RNA-seq datasets can be directly accessed through the ArrayExpress website by accessing https://www.ebi.ac.uk/arrayexpress/experiments/ followed by the ArrayExpress experiment number, i.e., https://www.ebi.ac.uk/arrayexpress/experiments/E-MTAB-2706/.

### Metanalysis of cancer survival hazard ratios

Overall survival trends expressed as hazard ratios (HR) for 80 unique human cancer studies representing 15 cancer types were obtained from the online databases Prognoscan (Mizuno et al., 2009) and PROGgene (Goswami and Nakshatri, 2013). For each PROGgene study (*n* = 68) the sample population was bifurcated at the median into high- and low-TAAR1 expression groups. Study data obtained from Prognoscan (*n* = 12) utilized a minimum *p*-value approach to determine the point of bifurcation into high and low expression groups. Briefly, the HR obtained for each study can be explained as the ratio of events (deaths) in the high TAAR1 expression group to events in the low TAAR1 expression group. To perform the meta-analysis after data collection all HR values were log-transformed to normalize values around zero to enable the calculation of subgroup averages and then back transformed to produce the average HR value. Forest plots were created using the R package ggplot2.

### Transcription factor binding site analysis

Transcription factor analysis of the TAAR1 5' untranslated region (input sequence: Human DNA sequence from clone RP11-295F4 on chromosome 6, complete sequence, GenBank: AL513524.8) was carried out with the transcription factor (TF) binding site predictor programs *LASAGNA-Search 2.0* (Lee and Huang, 2013, http://biogrid-lasagna.engr.uconn.edu/lasagna_search/) and *MatInspector* (Quandt et al., 1995; Cartharius et al., 2005, Bioinformatics) which is available as a free trial or part of a software suite from Genomatix available at https://www.genomatix.de/.

## Results

### Peripheral blood and platelets

The perpetual circulation of blood through the body provides a constant sentry of diverse immune cells capable of monitoring homeostatic alterations. This “sentinel principle” posits that blood cells reflect changes in overall physiology and is the basis for the practice of biomarker and liquid biopsy research that is foundational to the detection of many diseases (Burczynski and Dorner, 2006; Liew et al., 2006). The blood is composed of erythrocytes, leukocytes, platelets, and plasma. The plasma represents the largest volume of the blood. It is made up largely of water which includes various proteins, clotting factors, and other metabolic constituents. Erythrocytes represent the largest cellular portion of whole blood and predominantly function as oxygen carriers in cellular respiration. The remaining 1% of the blood is made up of leukocytes, which are the immune cells of the blood, and platelets, which facilitate blood clotting in response to injury and other homeostatic disruptions. Leukocytes are the mixed cell population of white blood cells consisting of all the immune cells of the blood and include cells of both the innate and adaptive response. More than a decade ago, TAAR1 mRNA was identified in circulating human leukocytes using RT-PCR (D'Andrea et al., 2003; Nelson et al., 2007).

Here, we utilized the NCBI GEO Profiles database (Barrett et al., 2013) to perform a search of TAAR1 RNA expression in blood, which revealed detectable expression in all of the 20 datasets obtained, representing blood from humans, rhesus monkeys, and mice. Accession numbers and references for expression datasets in whole blood, platelets, and PBMCs are summarized in Table 1. Accordingly, TAAR1 RNA is present in leukocytes and TAAR1 RNA is consistently detected in whole blood.

**Table 1:** Peripheral blood, platelets, and PBMC RNA expression datasets.

**Reference**	**GEO accession**	**GEO profile ID**	**Species**	**TAAR1 expression**
**WHOLE BLOOD**				
Kupfer et al., 2013	GDS6177	132620011	Human	Positive
Kupfer et al., 2013	GDS4938	114175011	Human	Positive
Bienkowska et al., 2009	GDS5277	120320211	Human	Positive
Kwissa et al., 2014	GDS5093	112376811	Human	Positive
Wong et al., 2009	GDS4274	96240711	Human	Positive
Wynn et al., 2011	GDS4273	96184311	Human	Positive
Newell et al., 2010	GDS4266	85973511	Human	Positive
Krupka et al., 2012	GDS4259	85768653	Human	Positive
van Leeuwen et al., 2008	GDS3325	81580189	Human	Positive
Berisha et al., 2011	GDS3881	71184862	Human	Positive
Julià et al., 2009	GDS3628	62836833	Human	Positive
Dusek et al., 2008	GDS3416	56333511	Human	Positive
Dumeaux et al., 2006	GDS1412	13887989	Human	Positive
Vanderford et al., 2012	GDS4237	91318763	Rhesus	Positive
Sharma-Kuinkel et al., 2013	GDS5315	121855661	Mouse	Positive
Tsuge et al., 2014	GDS4854	105758811	Human	Positive (Partial)
Vierimaa et al., 2006	GDS2432	32521911	Human	Positive (Partial)
Pimentel-Santos et al., 2011	GDS5231	118388438, 118388437	Human	Negative
Parnell et al., 2013	GDS4971	108634838, 108634837	Human	Negative
Parnell et al., 2011	GDS3919	72699438	Human	Negative
**PLATELETS**				
Raghavachari et al., 2007	GDS3318	54111911	Human	Positive
Nilsson et al., 2011	GDS5181	116562814	Human	Positive
**PBMCs**				
Cheadle et al., 2012	GDS5499	127549637, 127549638	Human	Positive
Ramos et al., 2014	GDS5363	123464437, 123464438	Human	Positive
Arita et al., 2013	GDS4974	108745653	Human	Positive
Shi et al., 2014	GDS4882	106226711	Human	Positive
Teles et al., 2013	GDS4551	98821011	Human	Positive
Hinze et al., 2010	GDS4267	96127911	Human	Positive
Moncrieffe et al., 2010	GDS4272	86177011	Human	Positive
Papapanou et al., 2007	GDS3326	81602811	Human	Positive
Malhotra et al., 2011	GDS4147	80546111	Human	Positive
LaBreche et al., 2011	GDS3952	73875811	Human	Positive
Merryweather-Clarke et al., 2011	GDS3860	70483511	Human	Positive
Bouwens et al., 2010	GDS3704	65335812	Human	Positive
McHale et al., 2009	GDS3561	60938636	Human	Positive
Cai et al., 2014	GDS4966	108405411	Human	Positive (Partial)
Ciancanelli et al., 2015	GDS5626	129019876,	Human	Negative,
		129002378		Negative
Dawany et al., 2014	GDS4786	103069376,	Human	Negative,
		103051878		Negative
Sancho-Shimizu et al., 2011	GDS4540	98390876,	Human	Negative,
		98373378		Negative

#### Platelets

In addition to their classic role in clotting, platelets are also thought to play a role in the initial innate immune response (Palabrica et al., 1992; Opal, 2000; Esmon, 2004; Theopold et al., 2004). Neutrophil-derived signals augment the thrombocytic response and in turn can help isolate pathogens and inhibit their entry into the system circulation (Hickey and Kubes, 2009; Massberg et al., 2010). In light of the likely possibility of cross-talk between platelets and innate immune cells it is interesting to note that there are detectable levels of TAs present in human platelets, and intra-platelet concentrations are significantly decreased upon platelet activation (D'Andrea et al., 2003). These initial data suggested that the release of TAs in response to injury or immune cell signals could have a functional role in the innate immune response. Using NCBI GEO data, we found evidence that platelets express TAAR1 RNA. TAAR1 RNA is detectable in human (Raghavachari et al., 2007, GDS3318; Piccaluga et al., 2014, GDS5405, GDS5406; Risitano et al., 2012, GDS4659) and mouse (Wright et al., 2014; Lee et al., 2016, GDS5320; Paugh et al., 2013, GDS4821) platelets, suggesting a potential for a possible feedback mechanism in platelet function (Table 1). The presence of TAAR1 RNA in platelets suggests a potential for platelet expression of TAAR1 protein and a potential for responses generated by TAAR1 agonists. It is important to note, however, that RNAs present in anucleate platelets are derived from the progenitor megakaryocyte cells from which platelets are derived, and therefore the TAAR1 RNA detected may or may not be utilized by platelets to synthesize TAAR1 protein. Platelets do, however, possess the machinery needed to translate RNA to protein and as such further study of platelet protein expression is needed to determine whether this cell type can produce functional TAAR1 protein (Weyrich et al., 2009). With this caveat in mind, TAAR1 protein expression in platelets could be a contributory mechanism of psychostimulant-induced effects on platelet-mediated immune responses, and so further investigation is necessary to examine protein expression and effects of selective TAAR1-targeted drugs on platelet function.

#### PBMCs

Peripheral blood mononuclear cells (PBMCs) are a mixed subpopulation of leukocytes lacking the more dense, granulated polymorphonuclear (PMN) cells. PBMCs include the B- and T-cells of adaptive immunity, as well as the natural killer (NK), NK T-cells, macrophage and dendritic cells of monocytic lineage of the innate immunity. Analysis of PBMC gene expression is widely utilized to identify biomarkers for disease diagnosis for an array of pathological conditions. PBMC transcriptomics are used in inflammatory conditions such as arthritis (Boyle et al., 2003; Shou et al., 2006; Wong et al., 2016), in various cancers (Burczynski et al., 2005; Showe et al., 2009; Piccolo et al., 2015), and in numerous psychiatric conditions including depression (Mendez-David et al., 2013; Fan et al., 2015), schizophrenia (Lai et al., 2011), and bipolar disorder (Herberth et al., 2011). In lieu of diseased patient populations, pharmacological cellular activation with mitogens or foreign antigens *in vitro* is used to model immune challenge-mediated changes in cellular function and gene expression patterns. Cellular mitogen Phytohaemagglutinin (PHA) stimulation is a well-known model of cellular activation. PHA has been shown to upregulate TAAR1 mRNA from human PBMCs relative to low TAAR1 mRNA levels at rest (Nelson et al., 2007). Analogously, PHA also induced a significant increase in rhesus monkey PBMC TAAR1 protein from low baseline levels and this upregulation augmented TAAR1 agonist-induced PKA and PKC phosphorylation (Panas et al., 2012). These data suggest that TAAR1 may be present at very low levels in normal physiological states but is upregulated in activated states, suggesting that it may be necessary in downstream responses related to cellular activation. Supporting this concept, work by Sriram et al. (2016) showed that viral antigenic exposure by HIV-1 infection upregulates TAAR1 protein in human PBMCs and that upregulation is augmented by pretreatment with the TAAR1 agonist methamphetamine (METH). Importantly, TAAR1 activation with METH increases HIV-1 viral titers and replication (Sriram et al., 2016). These data suggest that upregulation and activation of TAAR1 may be a mechanism by which anti-viral immune processes may be diminished or viral fitness is altered directly. It is intriguing to speculate that these effects may also occur in response to upregulation of TAAR1 resulting from amphetamine-like psychostimulant abuse, imbalances in TAAR1 ligand availability due to neurotransmitter level dysregulation as in psychiatric diseases, or modulations to endogenous TA levels through diet or host-microbiome interactions.

With regard to viral infections, however, our search of NCBI GEO revealed expression array studies in which human PBMC samples were devoid of TAAR1 at baseline (Table 1). For example, neither influenza virus infection nor a deficiency in the IRF7 gene, an important mediator of antiviral defenses, altered TAAR1 expression (Ciancanelli et al., 2015, GDS5626). Similarly, data derived from another study indicates that PBMCs infected with HIV alone or with tuberculosis co-infection were devoid of TAAR1 expression (Dawany et al., 2014, GDS4786). Data from another study (Cai et al., 2014, GDS5030, GDS4966) indicates that human PBMCs infected with active or latent tuberculosis lacked detectable TAAR1 expression as determined by a detection call of absent; however, one positive signal was obtained for a single uninfected sample (Cai et al., 2014, GDS4966). Furthermore, TAAR1 expression in both normal human PBMCs and those infected with Herpes simplex (Sancho-Shimizu et al., 2011, GDS4540) was absent and did not respond to the bacterial antigen lipopolysaccharide (LPS) or activation of the single-stranded DNA receptor TLR7/8 suggesting an inability of these cellular activation pathways to alter TAAR1 expression. It is important to remember, however, that the array-based technology used to determine presence of expression in these GEO datasets relies on detection calls derived from computational algorithms, and as such an “absent” call does not necessarily mean that the transcript is truly absent (Oudes et al., 2005). As such, there is a need for more TAAR1-specific expression profiling through less ambiguous methods such as RT-PCR and immunological staining.

In contrast to these virally-induced infectious diseases, our search of NCBI GEO revealed that TAAR1 RNA is detectable in PBMCs of patients with the inflammatory or immunological diseases osteoarthritis (Ramos et al., 2014, GDS5363), juvenile idiopathic arthritis (Hinze et al., 2010, GDS4267; Moncrieffe et al., 2010, GDS4272), and multiple sclerosis (Malhotra et al., 2011, GDS4147). TAAR1 RNA is also present in human PBMCs isolated from gastric, liver, and pancreatic cancers (Shi et al., 2014, GDS4882), as well as breast cancer (LaBreche et al., 2011, GDS3952). TAAR1 RNA was also present, but not differentially expressed from controls, in datasets from pulmonary hypertension (Cheadle et al., 2012, GDS5499), interleukin-10 treatment (Teles et al., 2013, GDS4551), nickel exposure (Arita et al., 2013, GDS4974), and benzene exposure (McHale et al., 2009, GDS3561), and these data are summarized in Table 1.

Taken together, our analyses found that *TAAR1* expression in PBMCs varies between studies and significant modulation of expression has only been demonstrated *in vitro* in response to cellular activation or immunological challenge. It may be that TAAR1 functionality in the immune system may mediate alterations to immune cell maturation processes. Our analysis indicates a role for TAAR1 in PBMC-derived erythroid maturation. TAAR1 levels are significantly higher (two-tailed *t*-test, *p* = 0.05) in the earliest stage of erythropoiesis (*n* = 3) vs. the later 3 stages (*n* = 9) (Merryweather-Clarke et al., 2011, GDS3860). Activation of TAAR1 present in these early progenitor cells would predictably alter signaling cascades involved in the phenotypic development of these cells. Further investigation into the disparity of TAAR1 expression between maturation stages may lend insight into a mechanism by which erythrocytic pathologies develop.

### Granulocytes

Granulocytes, also referred to as polymorphonuclear leukocytes (PMN), are a type of innate immune cells that act as the first line of cellular defense due to their ability to be recruited to the site of infection through chemotaxis. When activated these cells migrate toward chemoattractants derived from pathogens or local macrophage (Amulic et al., 2012). Granulocytes include phagocytic neutrophils that act initially to engulf foreign invaders and eosinophils that function primarily in defense against parasitic infections. Basophils are the rarest type of granulocyte and function in the initial infection response, allergic reactions, and in the T-cell polarization necessary for the adaptive immune response (Parham, 2009). Granulocytes express gene transcripts for TAAR1 and another TAAR family member, the orphan receptor TAAR2. Both TAAR1 and TAAR2 are co-expressed in subsets of human PMN (Babusyte et al., 2013). Interestingly, expression of both TAAR1 and TAAR2 was required for the chemosensory migration of PMN toward TAs, as evidenced by non-functionality when either TAAR was knocked down (Babusyte et al., 2013). If replicable, these data suggest the possibility of TAAR1/TAAR2 signaling interactions and/or dimerization as a prerequisite for chemotaxis of PMN toward TAs. Further studies are necessary to confirm if TAAR1 and TAAR2 form functional dimers. Although studies reporting functional subsets for blood granulocytes are few, there is an emerging indication for subset-specific markers (Clemmensen et al., 2012; Pillay et al., 2012). As such, TAAR1 and TAAR2 may represent markers for a granulocyte subset capable of chemosensory migration to TAs and other TAAR1 (and potentially TAAR2) ligands. These data also raise the possibility that the potent TAAR1 agonist METH and other TAAR1-targeted compounds could act in the same manner to alter chemotactic activity of particular subsets of PMN. Potentially, drugs that selectively target TAAR1 could potentially be used to treat migratory granulocyte dysfunction. Our NCBI GEO analysis revealed array-based evidence to support TAAR1 expression in granulocytes (Lattin et al., 2008, GSE10246). Overall, expression data for mixed granulocytes, eosinophils, neutrophils, and mast cells revealed a consistent TAAR1 signal (Table 2). No basophil-specific samples were available from the GEO database, so our analysis could not determine basophilic TAAR1 expression. While the relatively low-abundance of basophils could make the detection of low-level TAAR1 difficult, their role in the initial immune response and T-cell function necessitate targeted expression analysis of this cell type. Babusyte et al. (2013) provides convincing evidence that TAAR1 is in fact present in granulocytes but does not specify cellular subsets, so cell-type specific investigation is needed. Our analysis also indicates that mast cells, which are primarily involved in the allergic reaction and histamine release, also express TAAR1 (Lattin et al., 2008, GSE10246; Ito et al., 2012, GDS4420; Geoffrey et al., 2006, GDS2742). Finally, as all the granulocytes profiled in these GEO datasets were normal cells, it would be interesting to investigate any changes in TAAR1 expression levels with cellular activation.

**Table 2:** Granulocyte RNA expression datasets.

**Reference**	**GEO accession**	**GEO profile ID**	**Species**	**TAAR1 expression**
**MIXED GRANULOCYTES**				
Lattin et al., 2008	GSE10246	N/A	Mouse	Positive
**MAST CELLS**				
Geoffrey et al., 2006	GDS2742	39436016	Rat	Positive
Ito et al., 2012	GDS4420	89473161	Mouse	Positive (Partial)
**EOSINOPHILS**				
Holmes et al., 2015	GDS5468	127249361	Mouse	Positive
Wen et al., 2014	GDS5289	120861890	Mouse	Positive
Petersen et al., 2012	GDS4422	89566161	Mouse	Positive
**NEUTROPHILS**				
Radom-Aizik et al., 2008	GDS3073	47890111	Human	Positive
Hsu et al., 2011	GDS3776	67720674	Mouse	Positive
Petersen et al., 2012	GDS4422	89566161	Mouse	Positive

### Monocytes

Monocytes represent a short-lived subset of leukocytes with phagocytic ability that matures into macrophages and dendritic cells. At the time of initial immunological challenge, these monocytic cells of the innate immune system are the first to arrive and provide a rapid response to engulf pathogens and induce inflammation. Local phagocytic macrophages possess receptors called pattern recognition receptors that can recognize conserved pathogen antigens. Phagocytic cells engulf invaders on site while also releasing cytokines and chemokines that induce inflammation and the recruitment of other innate immune cells such as neutrophils and antigen-presenting cells (APCs). APCs are a bridge between the innate and adaptive immune response, processing antigens and presenting them to helper T-cells (Th) cells that will in turn undergo clonal expansion and activate cytotoxic T-cells (Tc) and B-cells. The result of this process is pathogen-specific immunological memory consisting of clonal populations of T- and B-cells that recognize and react to the initial specific antigen expressed by the pathogen (Janeway, 2001; Mogensen, 2009; Turvey and Broide, 2010; Monie, 2017). Babusyte et al. (2013) reported that human monocytes demonstrate variation in TAAR subtype mRNA expression as 20% of monocytes screened did not express any of the TAAR genes. There was some level of monocytic expression of all TAAR genes except TAAR8, and the TAAR2 signal was particularly high in this cell type (Babusyte et al., 2013). Our analysis of transcriptomic data for TAAR1 expression in mixed monocytic cell samples obtained from NCBI GEO (*n* = 22) strengthen the argument for TAAR1 presence in these cells, as 72.7% of datasets with measurements for TAAR1 contained positive signals for expression above detection threshold. Table 3 includes complete references containing queryable GEO Profile ID and GDS accession numbers. Notably, the only other manuscript published to date explicitly describing TAAR expression in monocytic cell types found that primary mouse macrophage and dendritic cells are devoid of all nine TAARs and are unresponsive to LPS or mouse gamma-herpes virus. TAAR1 was similarly absent in freshly isolated bone marrow cells and maturing bone marrow-derived dendritic cells and macrophages (Nelson et al., 2007). However, in contrast to this study are the various RNA expression datasets that reveal detectable expression of TAAR1 in macrophage. A search of datasets containing a probe representing the TAAR1 gene yielded 34 results of which 24 contained macrophage samples positive for TAAR1 expression (Table 3). Nine of the remaining ten datasets had a signal for TAAR1 expression that fell below the threshold for detection. Notably, a TAAR1 signal was completely absent in only one of the 34 data sets (Table 3). Our analysis of a macrophage gene expression profile (Gleissner et al., 2010, GDS3787) revealed significant TAAR1 upregulation with *in vitro* exposure to CXCL4, a cytokine released by activated platelets that plays a role in T- and NK cell migration and angiostatic activity.

**Table 3:** Monocyte RNA expression datasets.

**Reference**	**GDS accession**	**GEO profile ID**	**Species**	**TAAR1 expression**
**MONOCYTES**				
Zaritsky et al., 2015	GDS6082	132462276	Human	Positive
Bergenfelz et al., 2015	GDS5819	131699876, 131682378	Human	Positive
Hou et al., 2012	GDS4825	104614241, 104614242	Human	Positive
Sun et al., unpublished	GDS3088	48293635	Human	Positive
Maouche et al., 2008	GDS3555	60701719	Human	Positive
Maouche et al., 2008	GDS3554	60614511	Human	Positive
Papapanou et al., 2007	GDS3326	81602811	Human	Positive
Schirmer et al., 2009	GDS3690	64988198	Human	Positive
Boomgaarden et al., 2010	GDS3676	64485211	Human	Positive
Mosig et al., 2008	GDS3668	64121011	Human	Positive (Partial)
Dower et al., 2008	GDS3499	58902411	Human	Positive (Partial)
Ancuta et al., 2009	GDS4219	90466711	Human	Positive (Partial)
Menssen et al., 2009; Kyogoku et al., 2013	GDS4890	113484911	Human	Positive (Partial)
Menssen et al., 2009; Kyogoku et al., 2013	GDS4889	113428511	Human	Positive (Partial)
Maouche et al., 2008	GDS3553	60587246	Human	Below Cutoff
Woszczek et al., 2008	GDS3469	57728411	Human	Below Cutoff
Wheelwright et al., 2014	GDS4860	112723411	Human	Below Cutoff
Rosas et al., 2014	GDS5060	111205861	Mouse	Below Cutoff
Konuma et al., 2011	GDS3997	75442861	Mouse	Below Cutoff
Schirmer et al., 2010; van der Laan et al., 2012	GDS3658	63728398	Human	Negative
**MACROPHAGES**				
Mabbott et al., 2013	GSE49910	N/A	Human	Positive
Zahoor et al., 2014	GDS5294	121029711	Human	Positive
Gleissner et al., 2010	GDS3787	68032211	Human	Positive
Thuong et al., 2008	GDS3540	65911811	Human	Positive
Lee et al., 2009	GDS3595	61836153	Human	Positive
Maouche et al., 2008	GDS3555	60701719	Human	Positive
Maouche et al., 2008	GDS3554	60614511	Human	Positive
Maouche et al., 2008	GDS3553	60587246	Human	Positive
Chang et al., 2008	GDS3258	52891211	Human	Positive
Woodruff et al., 2005	GDS1269	1269	Human	Positive
Sirois et al., 2011	GDS4232	91083411	Human	Positive
Wu et al., 2012	GDS4258	85690111	Human	Positive
Verway et al., 2013	GDS4781	02866053	Human	Positive
Zaritsky et al., 2015	GDS6082	132462276	Human	Positive
Khajoee et al., 2006	GDS2182	27351937	Human	Positive
Lattin et al., 2008	GSE10246	N/A	Mouse	Positive
Waugh et al., 2014	GDS5356	123165461	Mouse	Positive
Franco et al., 2014	GDS4941	107744861	Mouse	Positive
Petersen et al., 2012	GDS4422	89566161	Mouse	Positive
Severa et al., 2014	GDS5605	128145261	Mouse	Positive
Zigmond et al., 2014	GDS5668	130496090	Mouse	Positive
An et al., 2014	GDS5422	125715974	Mouse	Positive
Satpathy et al., 2014	GDS5413	125332490	Mouse	Positive
Satpathy et al., 2014	GDS54134	125369190	Mouse	Positive
Kuo et al., 2011	GDS4527	97860725	Mouse	Positive
Woods et al., 2009	GDS3670	64239261	Mouse	Positive
Rivollier et al., 2012	GDS4369	84624390	Mouse	Positive
Goodridge et al., 2007	GDS2686	37829561	Mouse	Positive
Woodruff et al., 2005	GDS1874	1874	Mouse	Positive
Koziel et al., 2009	GDS4931	107562411	Human	Positive (Partial)
Kazeros et al., 2008	GDS3496	58799611	Human	Positive (Partial)
Chen F. et al., 2011	GDS4432	92283261	Mouse	Positive (Partial)
Rosas et al., 2014	GDS5060	111205861	Mouse	Below Cutoff
Zhang, 2013	GDS5634	129339861	Mouse	Below Cutoff
Bok et al., 2009	GDS3549	60421061	Mouse	Below Cutoff
El Kasmi et al., 2007	GDS3190	50927861	Mouse	Below Cutoff
Yamamoto et al., 2007	GDS2944	44607561	Mouse	Below Cutoff
Comer et al., 2006	GDS2410	31741561	Mouse	Below Cutoff
Edwards et al., 2006	GDS2041	2041	Mouse	Below Cutoff
Shell et al., 2005	GDS1285	1285	Mouse	Below Cutoff
Irvine et al., 2009	GDS3686	64840633	Human	Negative
**DENDRITIC CELLS**				
Bajwa et al., 2016	GDS6063	132375976, 132358478	Human	Positive
Salvatore et al., 2015	GDS5817	131566311	Human	Positive
Kerkar et al., 2014	GDS5384	124249353	Human	Positive
McGovern et al., 2014	GDS5349	122926242, 122926241	Human	Positive
Favila et al., 2014	GDS5086	112063411	Human	Positive
Kron et al., 2013	GDS4567	99378211	Human	Positive
Mezger et al., 2008	GDS2749	39640411	Human	Positive
Manel et al., 2010	GDS4225	90771211	Human	Positive
Kissick et al., 2014	GDS5631	129180561	Mouse	Positive
Balachander et al., 2015	GDS5665	130393390	Mouse	Positive
Bielinska et al., 2014	GDS5601	127969861	Mouse	Positive
Ippagunta et al., 2011	GDS5183	116636461	Mouse	Positive
Rivollier et al., 2012	GDS4369	84624390	Mouse	Positive
Frericks et al., 2006	GDS3504	82370750	Mouse	Positive
Weiss et al., 2010	GDS3813	68797261	Mouse	Positive
Fulcher et al., 2006	GDS2221	28328711	Human	Positive (Partial)
Macagno et al., 2006	GDS2216	28254211	Human	Positive (Partial)
Napolitani et al., 2005	GDS1249	10991311	Human	Positive (Partial)
Bosco et al., 2011	GDS3858	70380611	Human	Below Cutoff
Njau et al., 2009	GDS3573	61231811	Human	Below Cutoff
Ricciardi et al., 2008	GDS2750	39696811	Human	Below Cutoff
Szatmari et al., 2006	GDS2453	32923011	Human	Below Cutoff
Cisse et al., 2008	GDS3519	59489161	Mouse	Below Cutoff

### Lymphocytes

#### Natural killer and natural killer T-cells

Natural Killer (NK) cells are a type of cytotoxic lymphocyte important in the response to viral infection, the detection of tumor formation, and in the promotion of self-tolerance (Terunuma et al., 2008). 86.7% of NK cells isolated from human buffy coat samples had some level of mRNA for TAAR1,−2,−5,−6, or −9 that was detectable (Babusyte et al., 2013). NK cells isolated from mouse spleen also had detectable levels of gene transcripts for TAAR1,−2,−3, and −5 (Nelson et al., 2007), which is in agreement with our analysis of transcriptomic profiling (Table 4) of mouse spleen and pancreas (Fehniger et al., 2007, GDS2957; Sitrin et al., 2013, GDS4948, GDS4946). Array-based expression data exists to further support TAAR1 expression in NK cells (Table 4). A unique cell type that combines phenotypic characteristics of both NK cells and T-cells, termed NKT-cells, possess enhanced killing ability and have displayed significant antitumor function in preclinical studies (Schmidt-Wolf, 1991; Kim et al., 2007). Our analysis reveal TAAR1 RNA expression is detectable in mouse NKT-cells (Verykokakis et al., 2013, GDS5602), and therefore a targeted analysis of TAAR1 presence and function in this unique cell type is needed.

**Table 4:** Lymphocyte RNA expression datasets.

**Reference**	**GEO accession**	**GEO profile ID**	**Species**	**TAAR1 expression**
Mabbott et al., 2013	GSE49910	N/A	Human	Positive
**NATURAL KILLER CELLS**				
Sitrin et al., 2013	GDS4948	114385090	Mouse	Positive
Sitrin et al., 2013	GDS4946	114348390	Mouse	Positive
Stegmann et al., 2010	GDS4163	81241711	Human	Positive (Partial)
Fehniger et al., 2007	GDS2957	45000961	Mouse	Positive (Partial)
Dybkaer et al., 2007	GDS3191	50968911	Human	Below Cutoff
**NATURAL KILLER T-CELLS**				
Verykokakis et al., 2013	GDS5602	128016361	Mouse	Positive
**B-CELLS**				
Berglund et al., 2013	GDS5242	118877053	Human	Positive
Jelicic et al., 2013	GDS4863	112858353	Human	Positive
Lattin et al., 2008	GSE10246	N/A	Mouse	Positive
Kong et al., 2014	GDS5428	125919274	Mouse	Positive
Stolp et al., 2012	GDS4340	83554161	Mouse	Positive
Chen S. S. et al., 2011	GDS4178	82996861	Mouse	Positive
Chang et al., 2007	GDS2762	40097861	Mouse	Positive
Luckey et al., 2006	GDS1695	18100861	Mouse	Positive
Kim et al., 2006	GDS1807	20253311	Human	Below Cutoff
Fleige et al., 2007	GDS2805	41122650	Mouse	Below Cutoff
Patke et al., 2006	GDS2408	31695061	Mouse	Below Cutoff
Sato et al., 2005	GDS1467	14578761	Mouse	Below Cutoff
Garaud et al., 2011	GDS4193	95490311	Human	Negative
**T-CELLS**				
Palau et al., 2013	GDS5260	119509076, 119491578	Human	Positive
Sanda et al., 2013	GDS4754	101686911	Human	Positive
Twiner et al., 2008	GDS3433	56890700	Human	Positive
Twiner et al., 2008	GDS3429	56743700	Human	Positive
Villarroya-Beltri et al., 2013	GDS5639	129530637	Human	Positive
Lattin et al., 2008	GSE10246	N/A	Mouse	Positive
Trandem et al., 2011	GDS4217	90374190	Mouse	Positive
Jabeen et al., 2013	GDS5343	122668612	Mouse	Positive
Jabeen et al., 2013	GDS5291	120914412	Mouse	Positive
Heinemann et al., 2014	GDS5166	116131590	Mouse	Positive
Rudra et al., 2012	GDS5164,	116106850	Mouse	Positive
Chang et al., 2011	GDS4795	103262982	Mouse	Positive
West et al., 2011	GDS4555	98989561	Mouse	Positive
Kim et al., 2012	GDS4554	98947990	Mouse	Positive
Yang et al., 2011	GDS4572	94508161	Mouse	Positive
Yang et al., 2011	GDS4434	92366461	Mouse	Positive
Trandem et al., 2011	GDS4217	90374190	Mouse	Positive
Lee et al., 2012	GDS4334	88072790	Mouse	Positive
Sharma et al., 2011	GDS4333	88031161	Mouse	Positive
Pomi et al., 2011	GDS4373	84767690	Mouse	Positive
Johnson et al., 2012	GDS4355	84078490	Mouse	Positive
Kitoh et al., 2009	GDS3577	61386561	Mouse	Positive
Fontenot et al., 2005	GDS1113	10303961	Mouse	Positive
Fang et al., 2012	GDS4363	84350161	Mouse	Positive (Partial)
Kang et al., 2011	GDS3840	69765961	Mouse	Positive (Partial)
Setoguchi et al., 2009	GDS3566	61025461	Mouse	Positive (Partial)
Zhang et al., 2007	GDS3222	51651861	Mouse	Positive (Partial)
Kakoola et al., 2014	GDS5020	109918461	Mouse	Positive (Partial)
Fernandez et al., 2009	GDS4719	101630511	Human	Below Cutoff
Fernandez et al., 2009	GDS4188	95294411	Human	Below Cutoff
Wang et al., 2007	GDS2883	43226211	Human	Below Cutoff
Ndolo et al., 2006	GDS2164	26955811	Human	Below Cutoff
Kakoola et al., 2014	GDS5019	109871961	Mouse	Below Cutoff
Kakoola et al., 2014	GDS5018	109825461	Mouse	Below Cutoff
Su et al., 2005	GDS2717	38629261	Mouse	Below Cutoff
Zeng et al., unpublished	GDS1030	9277550	Mouse	Below Cutoff
Eom and Choi, 2010; Eom et al., 2014	GDS3783	67913172	Human	Negative

#### T-cells

T-cells are a lymphocytic subset of immune cells that are important in cell-mediated immunity. Stimulation with specific factors differentially activates T-cells to illicit the development of specific regulatory, effector, and helper functions and these phenotypes in turn shape the nature of the immune response (Barnes, 2011). Identifying physiological perturbations in T-cell function is crucial to understanding possible TAAR1 function in the immune system. There is a lack of both agreement and availability of literature exploring TAAR1 expression and function in T-cells and to date the only three manuscripts published on the topic have yielded opposing results. Observationally, the potent TAAR1 agonist METH alters T-cell function through mitochondrial injury, oxidative stress, and alterations to cytokine production (Potula et al., 2010). More specifically, work by Sriram et al. (2016) found that METH treatment increases TAAR1 mRNA and functional protein expression in human T-cells associated with a METH-induced decrease in secretion of the proinflammatory cytokine IL-2 and altered cAMP production. Importantly, these METH-induced effects were TAAR1-dependent (Sriram et al., 2016) suggesting that TAAR1 is expressed in T-cells and is capable of altering T-cell function. The same study also reported that HIV-1 positive METH users displayed enhanced expression of TAAR1 protein in T-cells of the lymph nodes compared to non-users. Elucidating the biology of HIV-1 infection in the context of concomitant chronic drug abuse is clinically important as METH-induced immunomodulation in HIV-1 infection could have a significant impact on treatment modulation and HIV-1 pathogenesis (Boddiger, 2005). Overall, current data suggest an association of TAAR1 expression, HIV-1 infection and METH and therefore more study of this possible connection is warranted. Supporting the hypothesis of lymphocytic modulation by TAAR1 is the observation that T-cells expressing TAAR1 and TAAR2 treated with TAs *in vitro* displayed increased secretion of IL-4, a cytokine that stimulates the proliferation of T- and B-cells (Babusyte et al., 2013). It is important to note that this effect was reportedly due to TA interaction with both TAAR1 and TAAR2, as the effect was lost with siRNA knockdown of either receptor (Babusyte et al., 2013), and therefore exploration of TAAR1/TAAR2 interactions is needed to replicate and elucidate this effect. To the contrary of the work of Sriram et al. (2016) and Babusyte et al. (2013), mouse splenic T-cells were earlier reported as devoid of TAAR1, TAAR2, TAAR3, and TAAR5 (Nelson et al., 2007). Our analysis reveals that 62% (*n* = 29) of the available RNA expression array datasets obtained from GEO profiles containing a TAAR1 probe were positive for measurable gene expression in T-cells in all samples, and 86% of datasets included one or more samples with measurable TAAR1 expression (Table 4). Accordingly, there is sufficient data pointing toward the presence of TAAR1 to warrant further research of its expression, signaling, and function in T-cells.

#### B-cells

B-cells are the antibody secreting lymphocytes and have been shown to express TAAR1 and other TAAR family members. B-cells isolated from human blood express TAAR1 and TAAR2 as well as TAAR5,−6, and −9 at lower levels (Babusyte et al., 2013). Panas et al. (2012) observed expression of functional TAAR1 that exhibits METH-induced TAAR1-dependent PKA and PKC phosphorylation in immortalized rhesus monkey B-cells. As immortalized cells may exist in a state mimicking constant immune activation, these investigators sought to recapitulate the TAAR1-dependent PKA and PKC phosphorylation in primary rhesus PBMCs. Indeed, PHA-activated primary rhesus PBMCs have upregulated TAAR1 mRNA expression and display the same PKA and PKC phosphorylation when treated with METH; however TAAR2 expression was uninvestigated. Similar cellular signaling governs the TAAR1-dependent, METH-induced modulation of monoamine transporter kinetic and internalization functions, discussed elsewhere (Miller et al., 2005; Xie and Miller, 2007, 2009). Notably, mouse B-cells were shown to express mRNA for TAAR1-4 at a moderate level and low-level expression was apparent for TAAR5-9 (Nelson et al., 2007). B-cells play a critical role in allergic inflammation by synthesizing and secreting antibodies such as IgE. TAs were demonstrated to induce secretion of IgE in purified human B-cells in a TAAR1/TAAR2-dependent manner (Babusyte et al., 2013). The ability of TAAR1 and TAAR2 co-expression to trigger IgE secretion in B-cells in response to TAs represents a new mechanism by which TAs directly alter immune cell function, and also raise the possibility that selective TAAR1 compounds may act similarly.

Our analysis of complementary GEO datasets confirmed B-cell TAAR1 expression in all but one (*n* = 13) dataset obtained (Table 4), supporting the expression of TAAR1 in this cell type.

It is interesting to note that TAAR1 expression in B-cells may vary based on maturation stage, as we had previously speculated for PBMC-derived erythroid progenitor cells. Retrospective analysis carried out with the NCBI GEO “Analyze DataSet” tool of the 2006 study of Luckey et al., 2006, GDS1695) revealed that TAAR1 transcripts are significantly higher in plasma B-cells than in more mature memory B-cells (two-tailed *t*-test, *p* ≤ 0.05).

### Cellular distribution of TAAR1 in the neuroimmune system

Neuronal expression of TAAR1 is documented in human dopaminergic brain regions including the ventral tegmental area, substantia nigra, hippocampus, amygdala, and other major regions (Borowsky et al., 2001; Espinoza et al., 2015). The interface between the immune system and the brain, commonly referred to as the neuroimmune system, is distinct in both its cellular population and physical permeability. The resident immune cells of the brain consist of astrocytes, microglia, and a unique type of macrophage that inhabits the perivascular and subarachnoid space (Engelhardt et al., 2017). The brain's lack of native T- and B-cells that make up the adaptive immune response in the periphery is due to a largely impenetrable physical barrier between the tissue of the brain and the blood circulation, known as the blood brain barrier (BBB).

#### Astrocytes

Astrocytes make up the largest population of brain cells and represent a dynamic component of neurological homeostasis, cell signaling, and immunological responses (Schubert et al., 1997; Ransohoff and Brown, 2012; Bazargani and Attwell, 2016). Some of the most critical astrocytic functions are perturbed by METH, namely disruption of the blood-brain barrier (Kousik et al., 2012; Ramirez et al., 2012; Northrop and Yamamoto, 2015; Turowski and Kenny, 2015) and glutamate clearance functionality (Cisneros and Ghorpade, 2014a). The role of TAAR1 in astrocytes was addressed by Cisneros and Ghorpade (2014a,b), who showed that TAAR1 was both present and functional in primary human astrocytes and signaled through cAMP. Importantly, TAAR1 mRNA and protein was upregulated by both METH and HIV-1 exposure. METH and/or HIV-1 treatment also increased the nuclear localization of TAAR1. Even more interesting was the apparent synergistic upregulation with combinatorial METH/HIV-1 treatment, observed in both immunological staining of protein as well as by assessment of mRNA expression. TAAR1 activation functionally altered the activity of the glutamate transporter EAAT-2, signifying the ability of TAAR1 agonists to modulate extracellular glutamate and therefore potentially mediate excitatory neurotoxicity.

Our analysis (Table 5) revealed that TAAR1 RNA is present in mouse forebrain astrocytes (Lau et al., 2012, GDS3944), normal human astrocytes (Grzmil et al., 2011, GDS4467), and epidermal growth factor-treated rat astrocytes (Liu et al., 2006, GDS2146). TAAR1 is also expressed in the human astrocyte cell line U251 (Lin et al., 2015, GDS6010) and embryonic stem cell (ESC) line H9-derived astrocytes (Lafaille et al., 2012, GDS4538). Another microarray experiment by the same group assessed expression profiles in induced astrocytes deficient in the UNC-93B protein (GDS4669), which produces a phenotype unable to signal through the viral antigen recognition receptors TLR3, TLR7, and TLR9 (Casrouge et al., 2006; Tabeta et al., 2006). Interestingly, the UNC-93B deficient astrocytes expressed higher TAAR1 RNA levels than cells normally expressing the protein. Therefore, TAAR1 upregulation may occur as a compensatory mechanism when anti-viral immune processes are disturbed.

**Table 5:** Neuroimmune cell RNA expression datasets.

**Reference**	**GEO accession**	**GEO profile ID**	**Species**	**TAAR1 expression**
**ASTROCYTES**				
Mabbot et al., 2013	GSE49910	N/A	Human	Positive
Lin et al., 2015	GDS6010	132273437	Human	Positive
Lafaille et al., 2012	GDS4538	98288041, 98288042	Human	Positive
Lafaille et al., 2012	GDS4669	101498241, 101498242	Human	Positive
Simpson et al., 2011	GDS4135	80120811	Human	Positive
Grzmil et al., 2011	GDS4467	93316011	Human	Positive
Lau et al., 2012	GDS3944	73585774	Mouse	Positive
Liu et al., 2006	GDS2146	26366816	Rat	Positive (Partial)
Zhang et al., unpublished	GDS2919	44030411	Human	Below Cutoff
Mense, 2006	GDS1779	19624611	Human	Below Cutoff
Mense et al., 2006	GDS2215	28197811	Human	Below Cutoff
Sharma et al., 2007	GDS3366	82062161	Mouse	Below Cutoff
Takasaki et al., 2007	GDS2725	38937116	Rat	Below Cutoff
**MICROGLIA**				
Yoshino et al., 2011	GDS4151	80724853	Human	Positive
Lattin et al., 2008	GSE10246	N/A	Mouse	Positive
Dirscherl et al., 2010	GDS3613	62315661	Mouse	Positive

Our analysis of the dataset by (Simpson et al., 2011) (GDS4135) revealed that TAAR1 RNA expression is positively correlated with increasing Braak staging, a clinical measurement of the progression of Alzheimer's and Parkinson's disease. Conversely, our analysis of a dataset obtained from the BioGPS database in which normal mouse astrocytes were incubated with brain slices from a beta-amyloid overexpressing mouse model of Alzheimer's disease (Kurronen et al., unpublished, dataset available from http://ds.biogps.org/?dataset=E-GEOD-29317&gene=111174) revealed that TAAR1 RNA expression was reduced relative to cells incubated with normal brain slices, and this difference could not be accounted for by age or developmental stage. Affymetrix microarray technology utilizes “detection calls” as a method of determining whether a gene transcript is present and allows for correction of noisy probe sets or error (Archer and Reese, 2010). While datasets with “absent” detection calls cannot be considered positive, it is still interesting to note that TAAR1 RNA was still measurable in other astrocytic samples from six other datasets (Table 5). Complementary searches of similar Affymetrix microarray experiments in BioGPS (Wu et al., 2013) further confirmed GEO results for expression in mouse (Beckervordersandforth et al., 2010; Zamanian et al., 2012), and suggests a bias in astrocytic TAAR1 expression toward the subventricular zone and hippocampal region relative to the ventral encephalon and the olfactory bulb (Mireia and Helena, 2012, unpublished; dataset available from http://ds.biogps.org/?dataset=E-GEOD-36456&gene=111174). These data and numerous other datasets for human TAAR1 expression can be freely browsed at http://biogps.org/gene/134864.

#### Microglia

Microglial cells are a distinct lineage of macrophage derived from the yolk sac that exists exclusively in the brain. These innate immune cells act analogously to the bone-marrow derived macrophage in the tissues and periphery to survey the microenvironment and respond to pathogens via pattern recognition receptors and toll-like receptor binding activity (Ginhoux et al., 2010; Saijo and Glass, 2011). Moreover, microglial expression of major histocompatibility complex-I (MHC-I) and MHC-II make them capable of antigen presentation to T-cells, required for adaptive immune responses (Hauser and Knapp, 2014). Our analysis of datasets accessed from NCBI GEO Profiles revealed that TAAR1 expression is detectable in the human microglial cell line HMO6 (Yoshino et al., 2011, GDS4151) and in mouse BV-2 microglia (Dirscherl et al., 2010, GDS3613). Further analysis of the array-based study by Yoshino et al. (2011, GDS4151) revealed that ethanol treatment induced a 1.4-fold increase in TAAR1 expression in HMO6 microglia relative to DMSO control, suggesting a potential interaction between the ubiquitous drug of abuse and TAAR1 modulation of functioning in the brain's major first line of immunological defense. Microglial TAAR1 signaling has yet to be directly studied, but considerable overlap exists between the immune activated state and TAAR1 expression. The potent TAAR1 agonist METH enhances HIV-1 replication in microglia and *in vivo* exposure to MDMA causes rat microglia to become activated (Pubill et al., 2003; Liang et al., 2008). Thomas et al. (2004) noted that the transcription factors (TF) NFκβ, cFos, and AP-1 needed for microglial activation are also upregulated by METH. Intriguingly, our analysis of the TAAR1 promoter carried out with the TF binding site predictor program LASAGNA-Search 2.0 (Lee and Huang, 2013) revealed that these same TFs are predicted to bind in the upstream untranslated region of the TAAR1 promotor, and these data are in agreement with an analogous search we conducted utilizing the similar program MatInspector (Quandt et al., 1995; Cartharius et al., 2005, Bioinformatics). In regard to astrocytes and microglia, in addition to the TAAR1 expression described in the literature, our analyses indicate that TAAR1 RNA is present in these neuroimmune cell types. Aberrant microglial activation, signaling, and function may be due to monoamine excess. Increased levels of the neurotransmitters dopamine and norepinephrine paramount to sympathetic nervous system activation may act in a positive feedback loop to mimic and perpetuate the immunologically activated state of microglia and this prolonged cellular activation could lead to cellular damage through production of reactive oxygen species and neuroinflammation. Alternatively, pathological changes in endogenous agonists for TAAR1, as seen in psychiatric disorders, could underlie alterations in microglial functions. While these proposed mechanisms are in contrast they are not mutually exclusive; it is likely that these pathways are intimately connected in a sensitive network of microenvironmental surveillance and homeostasis in response to trace and monoamine levels.

### TAAR1 is widely expressed in tissues of immune organs

Extensive antibody-based protein expression data is freely available online from the Human Protein Atlas (HPA) at www.proteinatlas.org (Uhlén et al., 2015; Thul and Lindskog, 2017). This database was generated by probing various human tissues for all protein-coding genes and is cataloged in a searchable module, allowing for tissue-specific exploration of expression patterns. Results of a June 2017 query of the HPA for TAAR1 protein expression yielded detection of TAAR1 in bone marrow and immune organs that included the appendix, spleen, bone marrow, tonsils, and lymph nodes (Table 6). Importantly, TAAR1 protein has been detected at appreciable levels in sites of immune cell maturation and activation, namely germinal centers in both lymph nodes and tonsils. As germinal centers are the location of B-cell maturation and fine-tuning of the adaptive immune response these data suggest that TAAR1 may be important in the B-cell mediated response. Similarly, TAAR1 protein is highly expressed in both the white and red pulp of the spleen and tonsillar germinal centers. TAAR1 protein is also highly expressed in lymphoid tissue and glandular cells of the appendix as well as glandular adrenal cells. Protein expression is summarized in Table 6, and histological images and details for these data are available online at http://www.proteinatlas.org/ENSG00000146399-TAAR1/tissue/primary$+$data.

**Table 6:** The Human Protein Atlas TAAR1 protein expression data in immune system tissues.

**Immune tissue**	**Protein expression level (HPA)**
**APPENDIX**	
Glandular cells	High
Lymphoid tissue	High
**Bone marrow**	Medium
**LYMPH NODE**	
Germinal centers	Medium
Non-germinal centers	Low
**TONSIL**	
Germinal centers	High
Non-germinal centers	Medium
Squamous epithelial cells	Medium
**SPLEEN**	
White pulp	High
Red pulp	High

Our retrospective analysis of a microarray dataset from HIV-1 infected lymphatic tissues (Li et al., 2009, GSE16363) containing gene probes for TAAR1,−2,−3,−5,−8, and −9 obtained from NCBI GEO revealed expression of all TAARs in normal tissue and tissue in acute and asymptomatic stages as well as fully progressed AIDS. TAAR1 was expressed at the lowest level of all TAARs and TAARs−2,−3, and −5 were most abundant. To explore changes in transcript levels of TAAR1 and related TAARs over the course of infection we obtained the raw count data for all samples and calculated log2 fold-change values for each infection stage relative to control tissues. All TAARs were downregulated in the asymptomatic stage of HIV-1 infection and all TAARs except TAAR3 were upregulated in acute HIV-1 infection. It is interesting to note that for TAAR1, expression appears to increase upon initial infection, decrease once the asymptomatic phase is reached, and return to control-baseline levels with progression to AIDS. TAARs are not significantly altered in HIV-1 infection, but trend toward upregulation with acute infection and downregulation with asymptomatic infection, corresponding to active viral replication and latent infection progressions. This finding agreed with our analysis of another GEO dataset of Dengue virus-infected blood in which TAAR1 RNA expression levels were significantly lower in convalescent infection vs. active viremia (Kwissa et al., 2014, GDS5093). The apparent increase in TAAR1 RNA in periods of active viral replication suggest that its expression could be modulated by the presence of viral antigen. Similarly, the downregulation of TAAR1 seen in asymptomatic infection, which corresponds to a period of viral latency, suggests that TAAR1 could play a role in the viral life cycle.

Recent online compilation of open-source transcriptomic profiling of immunologically relevant cell types is available through the Reference Database of Immune Cells (RefDIC), http://refdic.rcai.riken.jp (Hijikata et al., 2007). Our query for the TAAR1 gene revealed detection in numerous immunologically relevant cell types in two microarray datasets for human and one for mouse immune cells, representing three different Affymetrix GeneChip platforms. The acquired dataset of the Affymetrix GeneChip Mouse Array included the raw log2 expression values for a single probe representing TAAR1 RNA levels in 19 different cell types. TAAR1 was found to be expressed at low levels in various mouse immune cells and expression varied within cell types. Highest expression levels were observed in B-cells, T-cells, dendritic cells, macrophages, and mast cells. While the majority of TAAR1 expression-level counts for all immune cell types tend to be low, the distribution of the outlier cell types with higher expression counts, namely macrophage, appear to be skewed toward the innate branch of the immune system. This is likely because of a disproportionate amount of macrophage samples, so although observationally interesting this finding is not definitive.

The online repository BioGPS (http://www.biogps.org) is another abundant source of processed and searchable microarray expression datasets (Wu et al., 2013). A retrospective analysis of three microarray datasets retrieved from BioGPS (Mabbott et al., 2013, available from http://ds.biogps.org/?dataset=BDS _00013&gene = 134864) representing 1,049 human cell samples revealed TAAR1 gene expression in various immune cells including astrocytes, peripheral blood cells, leukocytes, monocytes, macrophage, and neutrophils. TAAR1 was also detected in mixed lymphocytes and in the lymphocytic subsets T-cells, Pre-, Pro-, and normal B-cells, and NK cells. TAAR1 was also present in low-medium levels in pancreatic islets. Similarly, analysis of two microarray datasets retrieved from BioGPS representing 232 mouse cell samples revealed detectable TAAR1 RNA in myeloid progenitor cells, thymocytes, CD4+ and CD8+ T-cells, NK cells, B-cells, mast and dendritic cells, macrophage, granulocytes, microglia, lymph node, and bone marrow (Lattin et al., 2008; Wu et al., 2013, dataset available from http://ds.biogps.org/?dataset=GSE10246&gene=111174).

### TAAR1 in cancers

The potent downregulation of the tumor-promoting gene SPP-1 as a result of TAAR1 activation has been previously described in T-cells (Babusyte et al., 2013). Notably, SPP-1 upregulation is inducible by PKC activation, a signaling pathway triggered by TAAR1 agonists, and its expression is inversely related to cancer prognosis as tumors rich in the SPP-1 gene have an enhanced ability to grow and invade other tissues to ultimately metastasize (Wai and Kuo, 2007). While no data currently exists for TAAR1-specific effects on cancer progression, a literature search for TAAR1 agonists in cancer revealed a small subset of manuscripts describing amphetamine as a tumor-promoter. The potent TAAR1 agonist amphetamine has been linked to cancer pathology since at least the 1990s, when daily amphetamine injections were found to increase tumor incidence, growth and metastases in virally-induced cancers on rats (Freire-Garabal et al., 1992, 1998). Similarly, a more recent human study (Chao et al., 2008) found that recreational amphetamine use correlates with increased risk of Non-Hodgkin's lymphoma (NHL). Specifically, hazard ratios determined from the study predicted that patients with weekly or more frequent use of amphetamines were 1.75 times as likely to develop NHL vs. their control counterparts and that recent use increased that risk to 4.3 times. Patients with 3 years prior use were three times as likely to develop NHL as drug-free patients. In contrast to the cancer-promoting effects of amphetamine are those of the potent endogenous TAAR1 ligand, 3-Iodothyronamine (T1AM). T1AM is a derivative of thyroid hormone and has been shown to inhibit growth of cancerous cells *in-vitro*. Specifically, *in-vitro* incubation of MCF7 human breast adenocarcinoma cells or HepG2 heptocellular carcinoma cells with T1AM resulted in reduced proliferation in an MTT assay. Further, IC_50_ values of T1AM were twice as high for control human foreskin fibroblast cells (Rogowski et al., 2017).

**Figure 1:**
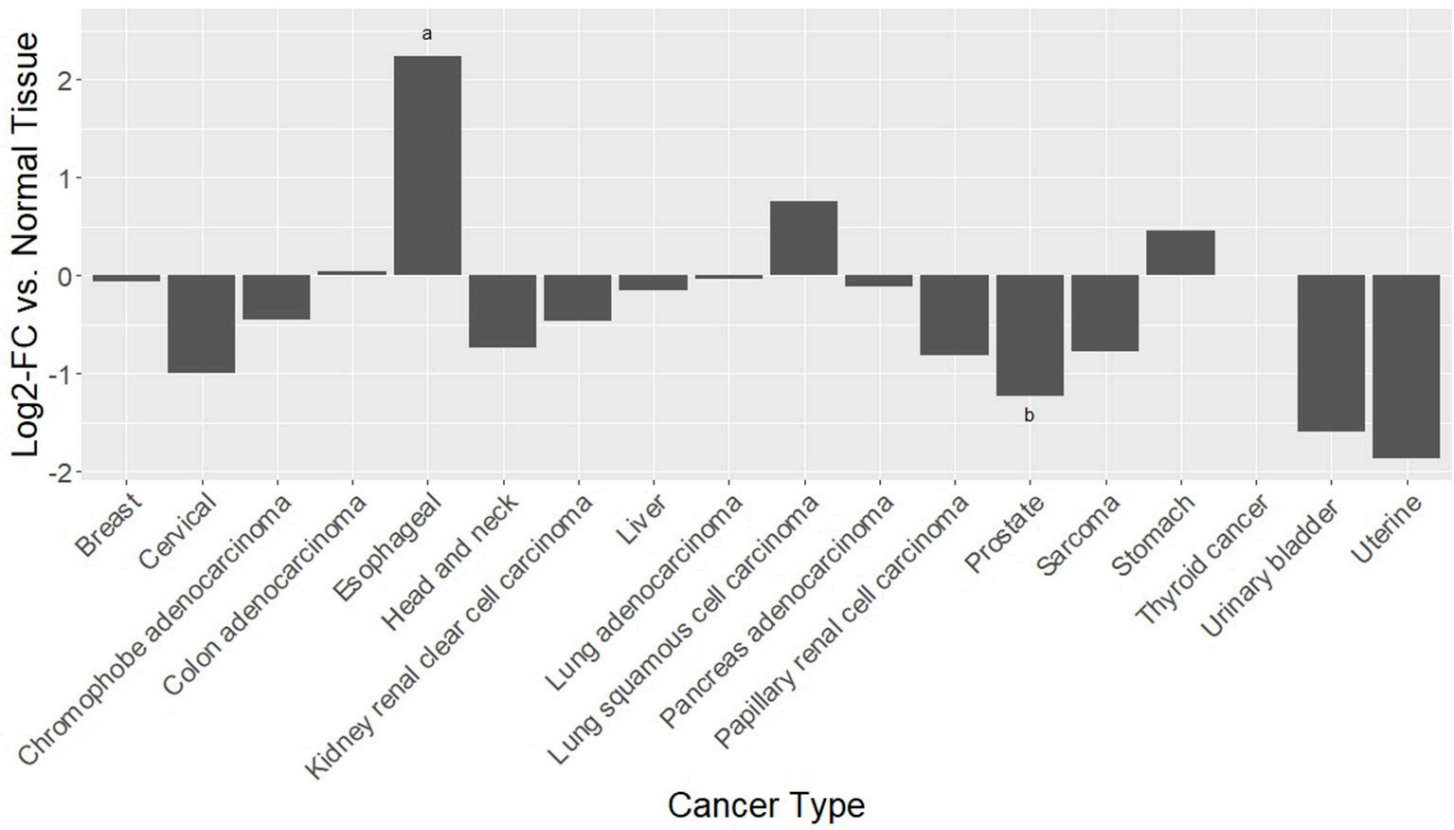
Differential RNAseq expression data for TAAR1 in human cancers. A TAAR1 mRNA transcript query in BioXpress (Wan et al., 2015) revealed that TAAR1 is upregulated in esophageal, lung, and stomach cancers, and downregulated in sarcoma, cervical, renal, kidney, liver, pancreas, pituitary, prostate, urinary, and uterine cancers. This differential expression is statistically significant in esophageal (^*^p = 0.023) and prostate (^**^p = 0.000043) cancers.

PKA, triggered by TAAR1 activation, is thought to act upstream in activation of the transcription factor NFKB (Bhat-Nakshatri et al., 2002). Chronic inflammation in the tumor microenvironment feeds forward to activate NFKB, which in turn perpetuates the inflammatory state that allows tumors to thrive (Karin, 2009). Constitutive activation of NFKB is associated with increased cancer risk and enhanced malignancy (Hoesel and Schmid, 2013). RNAseq data from 12 published cancer studies obtained from cBioPortal (Cerami et al., 2012; Gao et al., 2013) detected RNA for TAAR1, TAAR2, TAAR5, TAAR6, TAAR8, and TAAR9 in various cancers. A TAAR1 mRNA transcript query in BioXpress (Wan et al., 2015) revealed that TAAR1 is upregulated in esophageal, lung, and stomach cancers, and downregulated in sarcoma, cervical, renal, kidney, liver, pancreas, pituitary, prostate, urinary, and uterine cancers (Figure 1). This differential expression is statistically significant in esophageal (*p* = 0.023) and prostate (*p* = 0.000043) cancers. A gene query for TAAR1 in the online Catalog of Somatic Mutations in Cancer (COSMIC, Forbes et al., 2017) using the Genome Browser tool (https://cancer.sanger.ac.uk/cosmic/browse/genome) revealed that TAAR1 is overexpressed in at least 19 cancer types and in 16% of esophageal cancers. A similar gene-specific query in the Cancer RNA-Seq Nexus (Li et al., 2016) revealed that human TAAR1 is statistically differentially expressed in breast, bladder, cervical, lung, pancreatic, stomach, renal, and thyroid cancer (Table 7). Reexamining TCGA RNAseq data with respect to the TAAR1 gene (Figure 3) revealed varying RNA expression levels across cancer types. Overall, most cancer types displayed median TAAR1 RNA expression levels of zero, which is in agreement with the well-known phenomenon of TAAR1 expression detection being challenging (Liberles and Buck, 2006). Interestingly, cancer types containing a majority of samples without detectable TAAR1 expression also contained a number of samples with vastly varied expression levels, suggesting that TAAR1 expression varies in a patient-dependent manner even within a given cancer type. Lowest non-zero levels of TAAR1 RNA expression (maximum value for log2 TAAR1 expression < 1.5, which roughly represents values three times that of non-expression) were observed in Adrenocortical carcinoma [ACC, *n* = 80], Lymphoid Neoplasm Diffuse Large B-cell Lymphoma [DLBC, *n* = 48], Glioblastoma multiforme [GBM, *n* = 528], Glioma [GBMLGG, *n* = 696], Brain Lower Grade Glioma [LGG, *n* = 515], Pancreatic adenocarcinoma [PAAD, *n* = 185], Prostate adenocarcinoma [PRAD, *n* = 498], Stomach and Esophageal carcinoma [STES, *n* = 237], Testicular Germ Cell Tumors [TGCT, *n* = 150], Uterine Carcinosarcoma [UCS] *n* = 57. In summary, TAAR1 RNA was present at the lowest levels in cancers of the brain, pancreas, prostate, adrenal gland, stomach and esophagus, sex organs, and B-cells of the blood. As previously stated a large majority of samples in each cohort datasets lacked detectable RNA as represented by a median value of zero; however, median TAAR1 RNA levels were noticeably higher in the pan-kidney cohort representing KICH + KIRC + KIRP [KIPAN, *n* = 1020], Kidney renal clear cell carcinoma [KIRC, *n* = 607], Pheochromocytoma and Paraganglioma [PCPG, *n* = 179], and Skin Cutaneous Melanoma [SKCM, *n* = 470] cohorts. Therefore, TAAR1 RNA is most highly expressed in cancers of the kidney, skin, and neuro-endocrine cancers. It is interesting to note the substantial difference in TAAR1 RNA expression levels between the anatomically closely related adrenocortical cancers and the neuroendocrine cancers pheochromocytoma/paraglioma. An RNA-seq dataset containing 675 commonly used human cancer cell lines obtained from Array Express (Klijn et al., 2015, ArrayExpress experiment E-MATB-2706), 622 of which contained data for TAAR1 RNA expression, revealed that TAAR1 RNA is most highly expressed in the pancreatic somatostatinoma cell line QGP-1, lung carcinoid tumor cell line UMC-11, and the lung adenocarcinoma cell line VMRC-LCD. This dataset is summarized in Table 8 and directly available at https://www.ebi.ac.uk/arrayexpress/experiments/E-MTAB-2706/. Another Array Express RNA-seq dataset of long poly adenylated RNA and long non-poly adenylated RNA from ENCODE cell lines (Djebali et al., 2012, ArrayExpress experiment E-GEOD-26284) revealed that TAAR1 RNA is most highly expressed in the bone marrow neuroblastoma cell line SK-N-SH, normal human lung fibroblast cell line NHLF, human skeletal muscle cells, and the myoblast cell line HSMM. Further exploration of TAAR1 expression in cancers utilizing the COSMIC database revealed TAAR1 deletions in two cancer cell lines. a B-cell NHL subtype, mantle cell lymphoma, JEKO-1 cell line and malignant melanoma cell line Hs940.T. These data support the hypothesis that TAAR1 is modulated in cancers and therefore may serve a functional role in cancer physiology.

**Table 7:** Cancer RNA-seq Nexus datasets with differential TAAR1 RNA expression (adjusted *P*-value < 0.01) in differential expression analysis.

**Cancer type**	**Differential TAAR1 RNA expression**	**adjusted *P*-Value**
Adrenocortical carcinoma	No	
Breast carcinoma	Yes	0.000106887
Carcinoma of bladder	Yes	0.006578489
Cervical squamous cell carcinoma	Yes	0.000690563
Colon carcinoma	Yes	0.003349739
Colorectal carcinoma	No	
Cutaneous melanoma	No	
Endometrial carcinoma	No	
Esophageal carcinoma	No	
Glioblastoma	No	
Glioma	No	
Leukemia	No	
Liver carcinoma	No	
Lung adenocarcinoma	No	
Lung squamous cell carcinoma	Yes	0.004341319
Malignant neoplasm of colon with rectum	No	
Mesothelioma	No	
Ovarian serous adenocarcinoma	No	
Pancreatic carcinoma	Yes	0.008584279
Pheochromocytoma and paraganglioma	No	
Prostate carcinoma	Yes	0.00704065
Renal cell carcinoma	Yes	0.00095
Sarcoma	No	
Squamous cell carcinoma of the head and neck	No	
Stomach carcinoma	Yes	0.000878328
Testicular germ cell tumor	No	
Thyroid carcinoma	Yes	3.82E-06

**Table 8:** Cancer cell lines expressing TAAR1 RNA.

**Cell line**	**TPM**	**FPKM**
QGP-1, pancreas, pancreatic islet cell carcinoma	241	69
UMC-11, lung, lung carcinoid tumor	130	37
VMRC-LCD, lung, lung adenocarcinoma	33	8
NCI-H810, lung, non-small cell lung carcinoma	24	6
NCI-H1092, lung, small cell lung carcinoma	21	6
BEN, lung, lung carcinoma	19	5
NCI-H2081, lung, small cell lung carcinoma	10	3
DMS 454, lung, small cell lung carcinoma	6	2
NCI-H889, lung, small cell lung carcinoma	5	2
NCI-H146, lung, small cell lung carcinoma	3	0.8
NCI-H820, lung, lung adenocarcinoma	2	0.6
PK-59, pancreas, pancreatic carcinoma	2	0.5
HCC1359, lung, large cell lung carcinoma	2	
SNU-16, stomach, gastric carcinoma	2	0.5
NCI-H716, caecum, cecum adenocarcinoma	1	
NCI-H2369, lung, mesothelioma	1	
NCI-H2122, lung, non-small cell lung carcinoma	1	
SW 780, urinary bladder, urinary bladder transitional cell carcinoma	1	
RPMI 2650, nasal septum, nasal septum squamous cell carcinoma	0.8	
KP-3, pancreas, pancreatic adenosquamous carcinoma	0.7	
OCI-LY-10, lymph node, B-cell lymphoma	0.7	
OV-90, ovary, ovarian papillary serous adenocarcinoma	0.5	
NCI-H727, lung, lung carcinoid tumor	0.5	
NCI-H2052, pleura, mesothelioma	0.5	
KARPAS-1106P, lymph node, B-cell lymphoma	0.5	
COR-L47, lung, small cell lung carcinoma	0.5	

*Baseline RNAseq analysis of 622 human cancer cell lines obtained from EMBL-EBI ArrayExpress tool. TPM, Transcripts Per Kilobase Million; FPKM, Fragments Per Kilobase Million*.

TAAR1 signaling may modulate tumor cell function to alter malignancy and tumor progression and therefore TAAR1-specific compounds could potentially have oncological therapeutic potential and represent a novel approach to modulating cancer physiology. Accordingly, our analyses of TAAR1 expression trends in overall survival cancer studies raises speculation of TAAR1 as a possible prognostic marker. Serendipitously during the writing of this review, the first manuscript to posit TAAR1 as a predictor of overall survival in cancer was published. In histologically-based experiments, Vattai et al. (2017) found TAAR1 expression to correlate with longer overall survival in early breast cancer.

**Figure 2:**
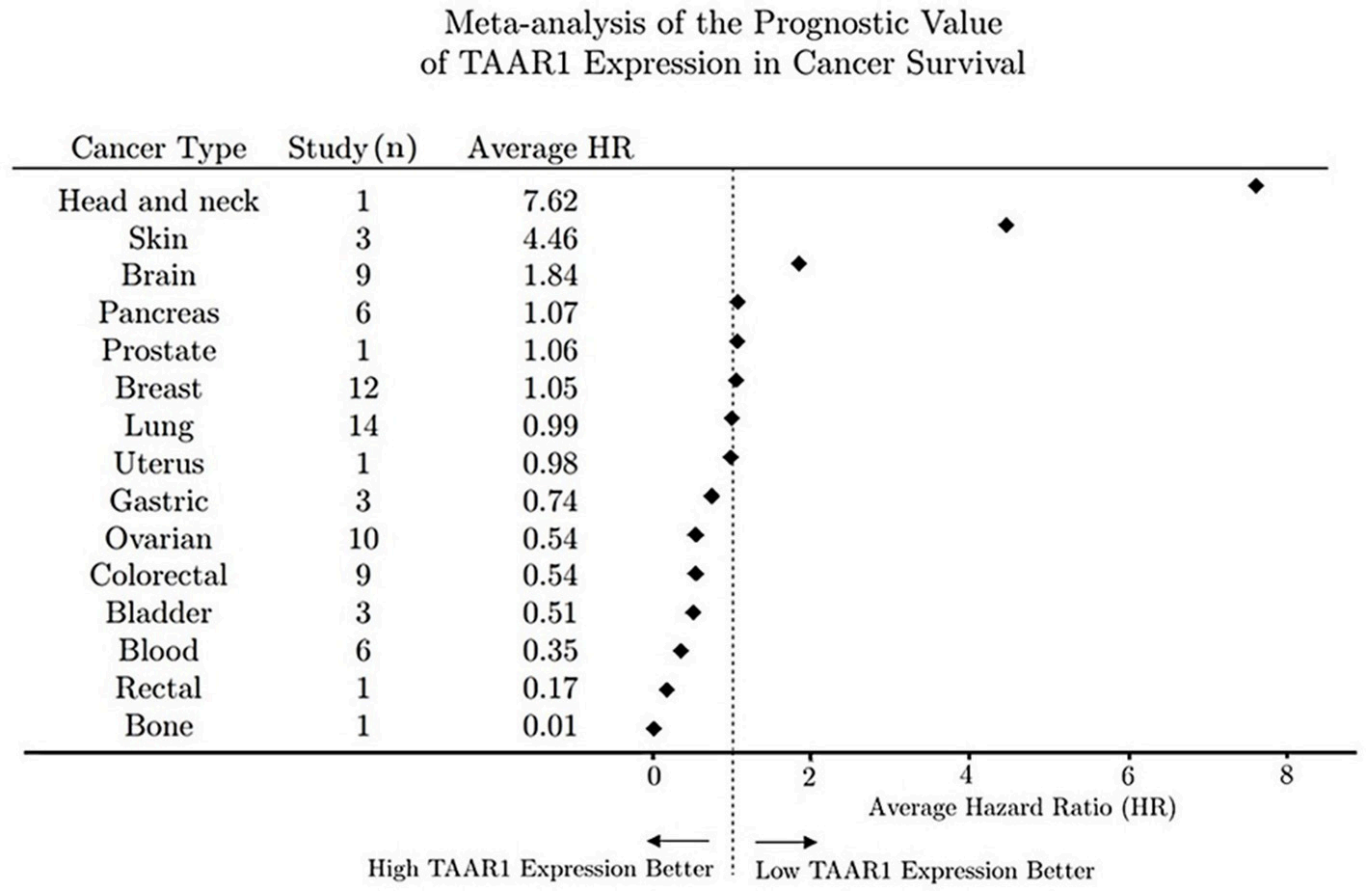
Meta-analysis of the prognostic value of TAAR1 expression in overall cancer survival. Forest plot of hazard ratios for survival in 80 human cancer studies representing 15 cancer types were obtained from open-source repositories (PROGgene *n* = 68, Prognoscan *n* = 12). Hazard ratios were determined for TAAR1 expression bifurcated into high and low expression and all HR values were log-transformed to normalize values around zero to enable the calculation of subgroup averages and then back transformed to produce the average HR value for each cancer type.

Overall survival trends expressed as hazard ratios (HR) for 80 unique human cancer studies representing 15 cancer types were obtained from the online databases Prognoscan (Mizuno et al., 2009) and PROGgene (Goswami and Nakshatri, 2013). For each PROGgene study (*n* = 68) the sample population was bifurcated at the median into high- and low-TAAR1 expression groups. Study data obtained from Prognoscan (*n* = 12) utilized a minimum *p*-value approach to determine the point of bifurcation into high and low expression groups. Briefly, the HR obtained for each study can be explained as the ratio of events (deaths) in the high TAAR1 expression group to events in the low TAAR1 expression group (Abel et al., 1984; Mizuno et al., 2009). To perform the meta-analysis after data collection all HR values were log-transformed to normalize values around zero to enable the calculation of subgroup averages and then back transformed to produce the average HR value denoted in Figure 2. An HR value of 1 denotes that survival was not different between the high and low expression groups, whereas an HR > 1 denotes that survival was poorer in the high expression group and conversely an HR < 1 means that survival was greater in the low expression group.

**Figure 3:**
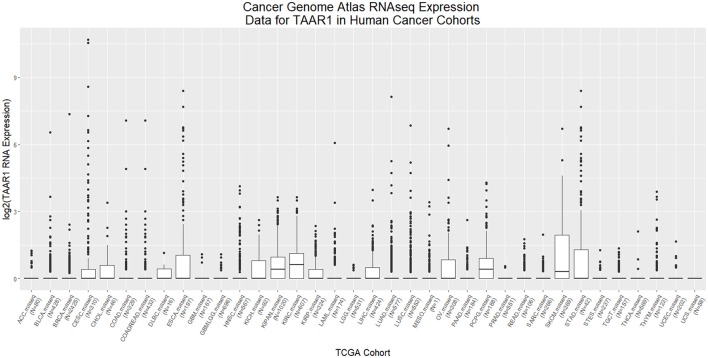
RNAseq analysis of TAAR1 expression in human TCGA cancer cohorts. RNAseq expression datasets representing cohorts from 36 cancer types were downloaded from The Cancer Genome Atlas (TCGA) using the R package RTCGA.rnaseq and values for the TAAR1 gene transcript were extracted, log2 transformed, and plotted with ggplot2. The minimal non-zero value for RNA expression levels was set to 1 to facilitate log2 transformation.

Our meta-analysis revealed that higher expression of TAAR1 correlates to longer median survival time in gastric, ovarian, colorectal, bladder, blood, rectal, and bone cancers. Conversely, lower TAAR1 expression correlated to longer median survival in head and neck, skin, and brain cancers (Figure 2). Breast cancer studies (*n* = 12) yielded an average HR of 1.05 which would suggest no differential effect of TAAR1 on cancer survival. Although these findings contradict the recent finding of Vattai et al. (2017), it is important to note that our meta-analysis did not account for progression stage, and as such a further analysis would be needed to directly compare our data with that of the early-stage breast cancer described in the 2017 manuscript. Overall survival analysis of a pancreatic cancer dataset (Grimont et al., 2015, GSE50827) stratified by cancer stage using PROGene V2 revealed that survival of stage IIB cancer that had spread from the pancreas to the lymph nodes is significantly higher (HR = 4.79, *p* = 0.025) vs. stage IIA cancer that lacks lymph node involvement, suggesting the possibility that TAAR1 may play a role in lymphoid cancer progression or pathways.

## Discussion

This review of current literature and meta-analyses of array-based evidence confirms that TAAR1 is expressed throughout various immune cell types and cancers. The ability of TAAR1 to signal in response to endogenous common biogenic amines and trace amines implicates the receptor as a mediator of aminergic regulation of immune function. Its ability to respond to exogenous amphetamine-like drugs implicates it as a modulator of at least some of the immunological actions of these drugs. Importantly, its ability to respond to dopamine implicates the receptor in the immunological action of drugs of abuse more generally, including drugs that do not directly bind to and/or activate the receptor directly, such as cocaine, but act through elevated dopamine levels. Additionally, its ability to be agonized or antagonized by newly developed synthetic drugs that are currently under development and investigation as psychiatric and addition therapeutics, respectively, mandate a greater definition of the immunological action of TAAR1-targeted drugs.

Our analyses further implicate TAAR1 as a logical target for studying the interplay between stimulant use and immune function. Modulation of neurotransmitter concentrations by METH and cocaine, for example, may act on TAAR1 either directly or indirectly to alter homeostatic immune cell signaling, in effect mimicking a prolonged state of immune activation, or by downstream changes in cellular function such as cytokine secretion, phagocytosis, and chemotaxis, for which there is emerging evidence.TAAR1-specific compounds could be efficacious therapeutics for infections and cancers, as well as valuable pharmacological tools for dissecting stimulant-induced changes and damage to immune function.

The expression of TAAR1 in various cancers and its functional significance is an intriguing yet largely unexplored area. Our analyses show that expression of TAAR1 is modulated in cancers, suggesting that TAAR1 serves a functional role in cancer physiology. We provide evidence for a differential pattern of cancer survival based on TAAR1 expression in multiple cancer types. The analyses indicate a potential prognostic value of TAAR1 detection in cancer survival that depends on the cancer type. Cancer pathologies differ widely and as such the observation that potentially protective effects of TAAR1 are type-specific supports the hypothesis that TAAR1 is utilizing cell type-specific signaling or collaborating with host cell receptor signaling in a cell type-specific manner to alter cellular function and cancer physiology.

The observational data gathered in previous decades linking amphetamine use to cancer progression needs to be revisited in light of the de-orphanization of TAAR1. As both illicit drug use and cancer incidence continue to increase globally it will be imperative to investigate TAAR1 signaling in cancer. TAAR1 signaling may play a role in the progression of many cancers, and accordingly TAAR1-specific compounds may serve as potential therapeutic additions to current clinical practices to improve survival. Pharmacologically elucidating TAAR1 signaling in cancer can lead to a better understanding of the mechanisms by which cancer eludes the immune system, and moreover, how drugs of abuse can contribute to cancer development and prognosis. Pharmacologically elucidating TAAR1 signaling, both generally with regard to TAAR1 as a protomer in receptor-receptor and/or receptor-protein complexes, or more specifically in cancer cellular phenotypes, will predictably lead to a better understanding of the mechanisms by which cancer alludes the immune system, and moreover, how drugs of abuse and other ligands for TAAR1 can contribute to cancer development, prognosis, and treatment. The current identification of TAAR1 as a marker in numerous cancers, potentially functional and therapeutically targetable, provides a logical direction for future studies on the effects of drugs targeting TAAR1. Our present analyses expand the spectrum of immune cells known to express TAAR1 and also provide a queryable roadmap for further investigation. The identification of TAAR1 across immune cell types presents an avenue for exploring both the role of TAAR1 in normal immune function as well as its potential role as a mediator or modulator of immune dysregulation. Immune susceptibilities in disease states and in particular those seen in drug users may involve aberrant TAAR1 regulation and function, and may be therapeutically targetable with drugs that interact with TAAR1.

## Author contributions

All authors contributed to the writing of the manuscript. LF performed all meta-analyses and compiled all data presentation.

### Conflict of interest statement

The authors declare that the research was conducted in the absence of any commercial or financial relationships that could be construed as a potential conflict of interest.
